# Unveiling New Horizons: Progress in the Management of Gastrointestinal and Hepatobiliary Cancer

**DOI:** 10.3390/cancers15184431

**Published:** 2023-09-06

**Authors:** Christian Sebesta, Harald Rosen

**Affiliations:** 1 Department of Hematology and Oncology, Klinik Donaustadt, 1220 Vienna, Austria; 2 Surgical Oncology, Sigmund Freud Private University Head of Surgical Oncology, 1020 Vienna, Austria

The field of gastrointestinal cancer research continues to make significant strides in understanding the complexities of these challenging diseases. In the realm of medical advancements, few areas have witnessed as much progress as the management of gastrointestinal (GI) and hepatobiliary cancer. With groundbreaking research that produces translationally evidence-based innovative treatments in the shortest possible time, the field has transformed, offering renewed hope to patients and healthcare professionals alike.

The advent of precision medicine has revolutionized cancer treatment, enabling tailored therapies based on a patient’s unique genetic profile. In GI and hepatobiliary cancer, targeted therapies have emerged as real game-changers. From anti-angiogenic agents to immune checkpoint inhibitors (with or without chemotherapy-backbones), these new treatment options have shown promising results in improving survival rates and quality of life for many patients.

In addition, the introduction of minimally invasive surgical techniques, such as laparoscopic and robotic-assisted operations, has equally transformed the landscape. These procedures offer numerous benefits, including an improved rate of R0-resections, reduced post-operative pain, faster recovery times, and the fewest possible functional limitations. As a result, patients can now undergo surgeries with decreased morbidity, empowering them to return to daily activities in a shorter time, as well as making them ready for further interdisciplinary therapeutic approaches.

The progress that has been achieved in the management of gastrointestinal cancer is remarkable in many ways. From advancements in screening and early detection to the integration of precision medicine and targeted (immuno-) therapies, patients diagnosed with GI or hepatobiliary cancers have much more promising prospects at present than were available at the beginning of this millennium. In a period that looks like a “blink of an eye” in the history of medicine, the first steps into a development that offers unimagined possibilities have been taken.

Impressively completed by minimally invasive surgical techniques and improved radiation therapy, multidisciplinary approaches have transformed the landscape of cancer care, offering patients better outcomes through increased response rates and extended survival times not only with regard to the progression-free survival (PFS), but encouragingly, also the overall survival (OS). All these achievements were out of reach just a few years ago.

The series of recent publications in this Special Issue of *Cancers* aims to shed light on various aspects of the diagnosis, treatment and management of colorectal, gastric, pancreatic and hepatobiliary cancers by illustrating the remarkable progress achieved in GI cancer management and the potential this holds for the future.

One noteworthy study, conducted by Rebecca Thonhauser and colleagues, explores the impact of induction chemotherapy with vascular endothelial growth factor (VEGF) inhibition on tumor response in patients with synchronously metastasized, potentially resectable colorectal cancer (mCRC) [1]. The researchers address an important gap in this knowledge by investigating the pathological tumor response of the primary tumor to induction chemotherapy.

Focusing on the cellular foundations, the research by Ana Montero-Calle and her team focuses on novel proteoforms derived from the alternative splicing of p53 and p63 proteins in colorectal cancer cells [2]. Their work introduces innovative diagnostic possibilities that may aid in the early detection of colorectal cancer.

The publication by Claudio Gambardella and collaborators delves into the use of biosynthetic mesh reconstruction after abdominoperineal resection for low rectal cancer [3]. The study highlights the significance of surgical healing and its correlation with oncological outcomes. The mesh reinforcement technique holds promise for improving postoperative recovery and enabling the timely initiation of adjuvant chemotherapy.

Advances in surgical techniques are also the focus of another recent publication in this issue of *Cancers*. Andrew J. Sinnamon and team introduce the Clockwise Anterior-to-Posterior—Double Isolation (CAP-DI) approach to portal lymphadenectomy in biliary tract cancer [4]. This innovative technique seeks to improve lymph node yield and outcomes in oncologic resection, addressing a critical aspect of biliary tract cancer management.

The newest diagnostic armentarium is the focus of the paper by Lucia Cerrito and coauthors, presenting a comprehensive review about the use of contrast-enhanced imaging in the management of intrahepatic cholangiocarcinoma [5]. Their work emphasises the evolving role of imaging techniques in diagnosing and managing this complex malignancy.

Another notable contribution is an exploratory assessment of nutritional evaluation tools as predictors of complications and sarcopenia in patients with colorectal cancer, conducted by Isabel M. Vegas-Aguilar and a team of dedicated researchers [6]. This study addresses the critical issue of malnutrition among CRC patients. Malnutrition not only diminishes overall survival rates and treatment effectiveness but also increases the risk of mortality. The authors hypothesize that the angle phase, a marker of nutritional status, could be linked to the risk of sarcopenia and cancer-related complications in CRC patients. By delving into the intricate relationship between nutritional status, sarcopenia, and cancer outcomes, this study paves the way for potential interventions that could enhance patient well-being and treatment outcomes.

Furthermore, the effect of perioperative blood transfusions and infectious complications on inflammatory activation and long-term survival following gastric cancer resection is a topic of significant importance. Noelia Puértolas and colleagues look into this critical aspect of gastric cancer management, highlighting the complex interplay between surgical procedures, immune response, and patient prognosis [7]. Their research underscores the need for a comprehensive understanding of the impact of perioperative factors on inflammatory markers and disease-free survival.

All these publications, as well as other contributions to this Special Issue of *Cancers,* collectively demonstrate and prove the multidimensional nature of gastrointestinal cancer research. From investigating novel nutritional assessment tools to understanding the implications of perioperative interventions for immune response and survival, research is making unstoppable progress toward improving the lives of cancer patients. The efforts and successes of networked scientific studies impressively and undoubtedly prove the importance of collaboration between researchers, clinicians and patients in the ongoing quest to unravel the complexities of gastrointestinal cancers.

In summary, this issue of *Cancers* aims to provide a glimpse into the exciting research dealing with various aspects of the genesis, nature, treatment, and management of gastrointestinal, hepatobiliary, and pancreatic tumors, and thereby hopes to contribute to the definition and assessment of the current state of modern cancer care in these selected specialties.
